# Anticancer potential of mebendazole against chronic myeloid leukemia: *in silico* and *in vitro* studies revealed new insights about the mechanism of action

**DOI:** 10.3389/fphar.2022.952250

**Published:** 2022-08-25

**Authors:** Julio Paulino Daniel, Felipe Pantoja Mesquita, Emerson Lucena Da Silva, Pedro Filho Noronha de Souza, Luina Benevides Lima, Lais Lacerda Brasil de Oliveira, Maria Elisabete Amaral de Moraes, Caroline de Fátima Aquino Moreira-Nunes, Rommel Mario Rodríguez Burbano, Geancarlo Zanatta, Raquel Carvalho Montenegro

**Affiliations:** ^1^ Laboratory of Pharmacogenetics, Drug Research and Development Center (NPDM), Federal University of Ceará, Fortaleza, Brazil; ^2^ Department of Biochemistry and Molecular Biology, Federal University of Ceará, Fortaleza, Brazil; ^3^ Department of Biological Sciences, Oncology Research Center, Federal University of Pará, Belém, Brazil; ^4^ Molecular Biology Laboratory, Ophir Loyola Hospital, Belém, Brazil; ^5^ Department of Physics, Federal University of Ceará, Fortaleza, CE, Brazil

**Keywords:** chronic myeloid leukemia, mebendazole, ABL1, allosteric inhibition, targeted therapy

## Abstract

Chronic myeloid leukemia (CML) is caused by constitutively active fusion protein BCR-ABL1, and targeting ABL1 is a promising therapy option. Imatinib, dasatinib, and nilotinib have all been shown to work effectively in clinical trials. ABL1 mutations, particularly the T315I gate-keeper mutation, cause resistance in patients. As a result, broad-spectrum ABL1 medicines are desperately needed. In order to screen potential drugs targeting CML, mebendazole (MBZ) was subjected to the *in vitro* test against CML cell lines (K562 and FEPS) and computational assays. The antiproliferative effect of MBZ and the combination with tyrosine kinase inhibitors (TKIs) was tested using end-point viability assays, cell cycle distribution analysis, cell membrane, and mitochondrial dyes. By interrupting the cell cycle and causing cell death, MBZ and its combination with imatinib and dasatinib have a significant antiproliferative effect. We identified MBZ as a promising “new use” drug targeting wild-type and mutant ABL1 using molecular docking. Meanwhile, we determined which residues in the allosteric site are important in ABL1 drug development. These findings may not only serve as a model for repositioning current authorized medications but may also provide ABL1-targeted anti-CML treatments a fresh lease of life.

## Introduction

Chronic myeloid leukemia (CML) is a hematopoietic stem cell disorder characterized by the accumulation of myeloid cells in the blood (Ning et al., 2020). CML is highly associated with chromosomal translocations, as the translocation between chromosomes 9 and 22 t (9; 22) (q34; q11), known as Philadelphia chromosome (Ph+), which is found in 95% of diagnosed cases (Haidary et al., 2021). The Ph + encodes for chimeric protein BCR-ABL1, which is a constitutively active tyrosine kinase (TK) linked to cellular growth, survival, and cell differentiation in CML (Amarante-Mendes et al., 2022).

Based on BCR-ABL1 importance to the CML progress, targeted therapy with tyrosine kinase inhibitors (TKIs), such as imatinib, has been developed (Deininger et al., 2005; Shanmuganathan et al., 2017). TK inhibitors compete with ATP for the ATP binding site of the receptor, affecting protein phosphorylation and thus significantly reducing intracellular signaling and CML progress (Rosti et al., 2017; Jiao et al., 2018). However, mutations in the ATP binding site leading to intrinsic and extrinsic mechanisms of drug resistance led to therapy failure, requiring the development of second and third generations of TKIs, such as nilotinib, dasatinib, bosutinib, and ponatinib to overcome the resistance in patients with CML (Pophali and Patnaik, 2016; Machado et al., 2021). Therefore, it is imperative to develop studies seeking new molecules or redirect already known drugs to target the BCR-ABL protein with potential application in the CML.

Drug repurposing (DR) is a pharmaceutical strategy for the discovery of new therapeutic applications to a drug already employed for another disease (Andresen and Gjertsen, 2017). DR reduces the time and costs to develop a new drug and become clinically available. For example, the development of a novel drug could take up to 15 years from lab to application with costs of around 2–3 billion US dollars (Nosengo, 2016; Cha et al., 2018). DR is highly beneficial for both pharmaceutical companies and patients for some reasons: 1) the time estimated for approval of a repositioned drug is two times (6 years) lower than that for a new drug; 2) DR reduced costs, making the studies cost-effective for the pharmaceutical industry and beneficial for patients; 3) DR reduced the chances of the development of side effects, as the drugs have already been employed for another disease (Nosengo, 2016). Therefore, the DR studies aim to discover new pharmacologic mechanisms and new molecular targets for known drugs to reach new applications (Gurova, 2009).

In this context, benzimidazoles (BZLs) are a class of heterocyclic compounds formed by the fusion of benzene and imidazole rings. BZLs’ first derivative was synthesized by Hobrecker in 1872, and since then, a wide range of pharmacological activities have been associated with these compounds (Pathare and Bansode, 2021). Mebendazole (MBZ) is an approved benzimidazole used in the clinical practice for the treatment of a broad spectrum of helminth infections by affecting tubulin polymerization. Moreover, MBZ has proved its antitumoral activity extensively in the lung, colon, medulloblastoma, melanoma, and gastric cancer, among other sites (Doudican et al., 2013; Nygren et al., 2013; Bodhinayake et al., 2015; Pinto et al., 2015, 2019; Guerini et al., 2019).

Recently, Li et al. (2019) have suggested MBZ to treat human acute myeloid leukemia. However, its effect on CML and in the BCR-ABL modulation remains unexplored. Therefore, this study aimed to better characterize the antileukemic potential of MBZ in resistant and non-resistant CML cell lines *in vitro*. We also sought to investigate the effect of the MBZ in BCR-ABL modulation by using *in silico* methods.

## Materials and methods

### Chemicals

Benzimidazole compounds mebendazole (MBZ) and albendazole (ALB) were dissolved in dimethyl sulfoxide (DMSO) and stored at −20°C, as well as the chemotherapeutic drugs traditionally used in CML, imatinib (Gleevec® 400 mg—Novartis) and dasatinib (Sprycel® 20 mg—Bristol-Myers Squibb).

### Cell culture

CML cell line K562 and its chemoresistant derivative (FEPS) were used in this study (Daflon-Yunes et al., 2013). Both were provided by Prof. Dr. Vivian Rumjanek, from the Federal University of Rio de Janeiro (UFRJ). The cells were cultured in the RPMI medium, supplemented with 10% fetal bovine serum and 1% streptomycin/penicillin. In addition, the FEPS cell line culture medium was supplemented with daunorubicin (466 nM final concentration) to promote and maintain its chemoresistance profile. Thus, the cell culture was kept in a 5% CO_2_ chamber at 37°C and constantly monitored under an inverted microscope.

### Alamar blue assay

The cell lines were seeded in 96-well plates at a density of 1 × 10^6^ cells/ml and then treated with a concentration–response curve (0–20 µM) to determine the inhibitory concentration at 50% activity (IC_50_) of MBZ and ALB, and imatinib and dasatinib (commercial kinase inhibitors), alone and in combination with MBZ. After 72 h of treatment (HT), Alamar blue solution (1:20 in RMPI culture medium without SBF) was added and the plate was placed in the culture incubator for 3 h. Then, the plates were read using a multi-mode microplate reader (BioTek Instruments) at a wavelength of 465/540 nm. After evaluating the cell viability, further experiments were performed using concentrations based on IC_50_ values. Combination index (CI) calculations provide a great quantitative result for analyzing how two distinct drugs interact: additive effect (CI = 1), synergism (CI 1), and antagonism (CI > 1). Thus, using the CompuSyn software and the Chou-Talalay method, the synergistic effects of imatinib, dasatinib, and mebendazole were determined (Chou, 2010). The resistance index (RI) was calculated by the ratio between the IC_50_ of each substance alone or in combination according to the following formula:

$$RI=\;\frac{IC50\;FEPS\;lineage}{IC50\;K562\;lineage}$$

### Cell cycle progression

Cells were seeded in 12-well plates at a density of 1 × 10^5^ cells/ml and kept for 24 h incubated with a 5% CO_2_ atmosphere at 37°C. After that, CML cell lines were treated with MBZ, ALB, imatinib, and dasatinib alone and in association based on IC_50_ for 72 h. Then, cells were fixed with 70% ethanol at 4°C overnight. After the cells were centrifuged at 1500 rpm for 5 min, the supernatant was removed, and cells were incubated with 0.1% Triton-X solution (Sigma-Aldrich) for 10 min at room temperature. After new centrifugation and supernatant removal, the cells were incubated in RNAse-free DNA solution (100 μg ml^−1^) for 20 min at 37°C. Then, propidium iodide (PI) was added directly into the RNAse solution at a final concentration of 50 μg ml^−1^. After a 1-h incubation, cells were centrifuged again to remove the supernatant and resuspended in ×1 PBS solution. Thus, 10,000 events were analyzed by flow cytometry (BD FACSverseTM).

### Cell membrane integrity and mitochondrial membrane potential

Cells were seeded in 12-well plates at a density of 1 × 10^5^ cells/ml and then treated with the compounds alone or in combination. After 72 h, the cells were then transferred to 1.5 ml tubes, centrifuged at 1500 rpm for 5 min, and the supernatant was discarded. Thereafter, Rhodamine 123 (Rho123, 10 μg ml^−1^) and PI (5 μg ml^−1^) in the RPMI medium were added. Cells were incubated for 35 min in a CO_2_ chamber, centrifuged (as mentioned earlier), and resuspended in 1x PBS solution, and 10,000 events per sample were analyzed in a flow cytometer (BD FACSVerseTM).

### *In silico* pharmacodynamic analysis

The Molinspiration server (http://www.molinspiration.com/cgi-bin/properties) was used to calculate a biological activity score for the six most important drug classes: GPCR ligands, kinase inhibitors, ion channel modulators, nuclear receptors, protease inhibitors, and enzyme inhibitors. The possible molecule targets were evaluated by prediction with the SwissTargetPrediction server (http://www.swisstargetprediction.ch/), by comparing the similarity of compounds with a library of 280,000 active compounds and more than 2,000 pharmacological targets from different organisms (Gfeller et al., 2014). Thus, the predicted targets were ranked according to a proprietary probability function of the system.

### Molecular structure preparation

Initially, the protein structures of ABL1 co-crystallized with ligands were obtained from the Protein Data Bank (PDB, https://www.rcsb.org/), identified by PDB ID 2HYY, 2GQG, and 5MO4, with the resolution of 2.4 Å, 2.4 Å, and 2.17 Å, respectively. In the ATP binding site of these structures, ligands imatinib (PDB ID: 2HYY), dasatinib (2GQG), and nilotinib (5MO4) were co-crystallized, while the 5MO4 structure is also complexed with the asciminib ligand in the allosteric site.

The protein structures were visualized and analyzed using PyMol 2.3.0 (https://pymol.org/) to remove water molecules, native ligands, and any other reagents present in the three-dimensional (3D) models. The online tool PDB2PQR 2.1.1 (https://server.poissonboltzmann.org/pdb2pqr) was used to set protein protonation at the physiological pH. Atomic charges were assigned using Kollman charges, and non-polar hydrogen atoms were removed from the structure.

False-positive (decoy) ligands were obtained using the DUDE generate tool (http://dude.docking.org/generate). The ligand protonation state was established for target protein pH crystallization using the Avogadro program version 1.2.0 (Avogadro Chemistry). The ligand structure was minimized with the “optimize geometry” tool, settled to 10,000 steps, MMFF94 force field, and “steepest descent” algorithm. Charge assignment was performed using the Gateiger model in AutoDock Tools 1.5.6.

### Molecular docking

Molecular docking was performed using AutoDock 4.2.6 (https://autodock.scripps.edu/download-autodock4/). Redocking simulations with a threshold of 2.0 Å were performed using structures 2HYY, 2GQG, and 5MO4 to dock ligands imatinib, dasatinib, nilotinib, and asciminib. The Lamarckian genetic algorithm was set to 2,500,000 energy evaluations per run, 150 individuals in a population, and a maximum of 27,000 generations per run. For each system, docking was performed six times, generating 900 poses per ligand. Results were clustered using AutoDock Tool 1.5.6, with a threshold of 2.0 Å. For each system, the pose belonging to the most populated cluster with the lowest calculated interaction energy was selected as the representative structure. Discovery Studio Visualizer 19.1.0 software (https://discover.3ds.com/discovery-studio-visualizer-download) was used for interaction visualization, classification, and identification of participating residues.

### Statistical analysis

The quantitative data from the cellular *in vitro* experiments were analyzed by the mean and standard deviation (SD) calculations of three independent experiments. Data were compared by analysis of variance (ANOVA) followed by the Bonferroni/Tukey test, or their nonparametric equivalents, at a 95% significance level (*p* < 0.05) to check for different groups and experimental conditions. GraphPad Prism® 8.0 software was used for statistical analysis.

## Results

### Antiproliferative activity of mebendazole against chronic leukemia cell lines

MBZ showed a highly cytotoxic effect against K562 cell lines, with an IC_50_ at a concentration of 104.3 nM, which is 30% lower than imatinib effects (134.6 nM) and around 400% lower than ABZ concentration (486.4) to reach IC_50_. MBZ presented an IC_50_ at a concentration of 1.9 µM against a chemoresistant cell line (FEPS), which is five times lower than the concentration of imatinib (9.6 µM) (Table 1). Dasatinib showed the best IC_50_ value for both sensitive and resistant cell lines, which is improved in combination with MBZ but only in the K562 cell line (Table 1). It is worth noticing that MBZ presented an synergic effect with both dasatinib and imatinib (Table 1). The association of imatinib + MBZ leads to an IC_50_ at a concentration of 49.74 and 591.1, respectively, to K562 and FEPS cell lines and potent cytotoxicity with a resistance index of 18, the lowest RI among the tested groups (Table 1). Moreover, the combination index (CI) was determined to identify synergism, additive effect, or antagonism. After applying the Chou-Talalay method, a strong synergic effect was identified between mebendazole association with imatinib (CI = 1.04 × 10^−4^) or dasatinib (CI = 0.01205) in K562 cell line. The same effect was observed for the chemoresistant cell line FEPS. MBZ in association with imatinib (CI = 0.1639) or dasatinib (CI = 0.9418) had a combination index <1.

**TABLE 1:** Cytotoxic activity of imatinib, dasatinib alone, and in combination with mebendazole against chronic myeloid leukemia cell lines.

	IC_50_ nM (CI95%)		
Compound	K562	FEPS	RI
MBZ	104.3 (60.3–180.5)	1,917.0	18
ABZ	486.4 (199.0–1,189.0)	ND	ND
Imatinib	134.6 (71.62–252.9)	9,661.0 (1,101.0–84,780.0)	72
Dasatinib	0.15 (0.07–0.32)	33.54 (6.61–17.3)	224
Imatinib + MBZ	49.74 (25.46–97.19)	591.1 (326.1–1,072.0)	12
Dasatinib + MBZ	0.06 (0.014–0.223)	411.3 (138.7–1,219)	6855

RI, resistance index; CI, combination index; ND, not determined.

### Mebendazole plus BCR-ABL inhibitors blocked the cell cycle and induce cell death

**FIGURE 1:**
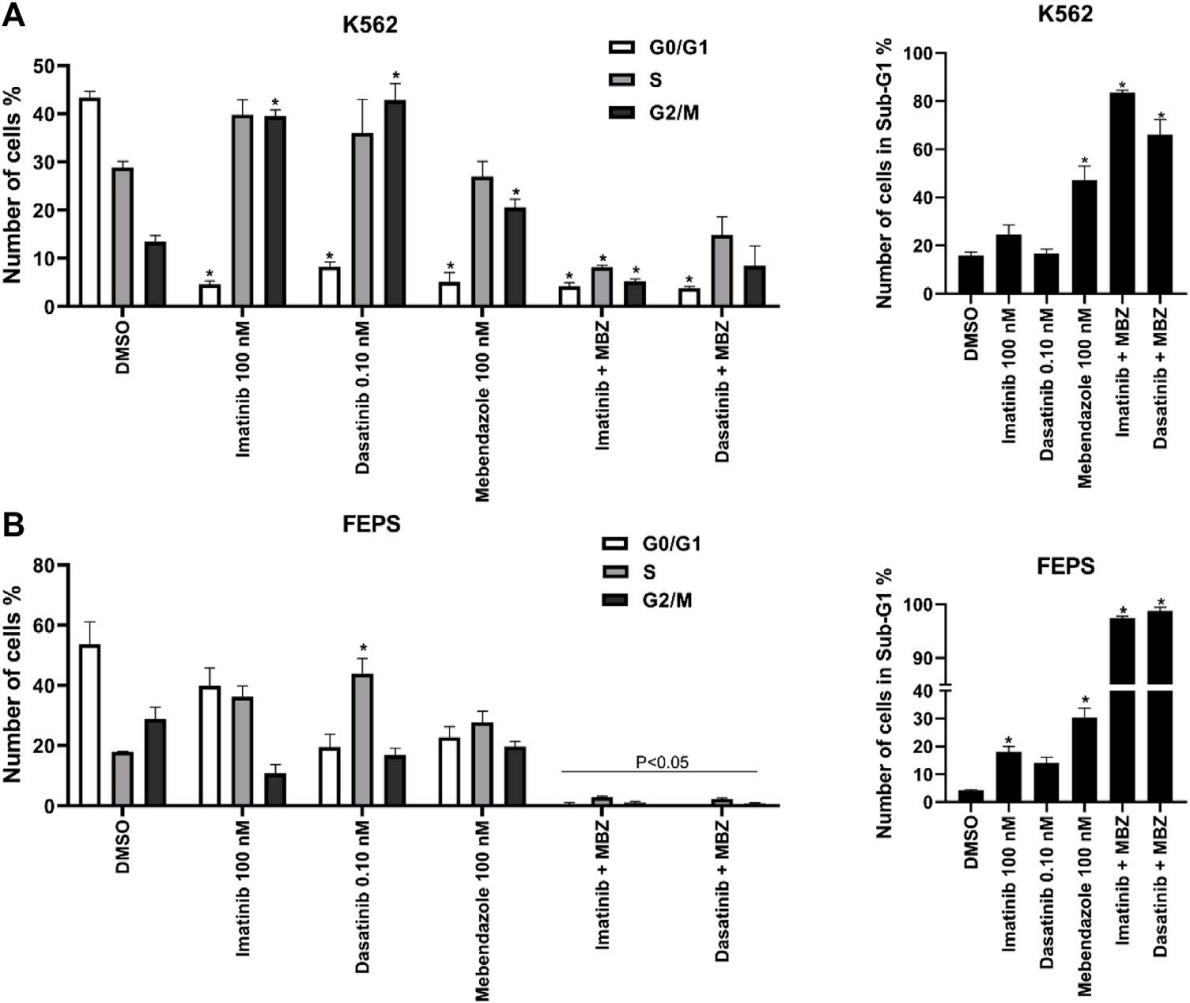
Mebendazole (MBZ) alone and in combination with ABL1 inhibitors induce cell cycle arrest in the chemosensitive and chemoresistant CML cell lines. Cell cycle distribution after the treatment with imatinib (100 nM), dasatinib (0.10 nM, mebendazole (100 nM) in K562 cell line **(A)** and FEPS cell line **(B)**. Data are represented as the mean standard deviation of three independent experiments. Significant differences among the groups were tested by the two-way ANOVA, followed by the Turkey multiple comparison test.

To shed light on the antiproliferative effects of MBZ alone or in combinations with ABL1 kinase inhibitors due to cytostatic effect, cell cycle, cell membrane, and mitochondrial integrity analysis were performed. Imatinib and dasatinib used in K562 showed statistical difference (*p* ≤ 0.01 and *p* ≤ 0.0001) in the number of cells with fragmentation profiles when compared with the control group. Similarly, the combination dasatinib + MBZ also was statistically different from treatments with the drugs alone (*p* ≤ 0.001) (Figure 1A). Regarding the cell cycle, MBZ alone and its combination with imatinib reduced the percentage of cells in the G0/G1 phases, and increased the DNA fragmentation (Sub-G1 phase), as did the combination with dasatinib, which also reduced the number of cells in the S phase (*p* ≤ 0.0001, Figure 1A). Similarly, for FEPS, the percentage of fragmented cells in the treatment with MBZ alone and for the combinations was statistically different (*p* ≤ 0.0001) from that of the control and imatinib and dasatinib alone. In this case, the fragmentation was so high that it was practically not possible to determine the cells’ fraction in each cell cycle phase (Figure 1B).

**FIGURE 2:**
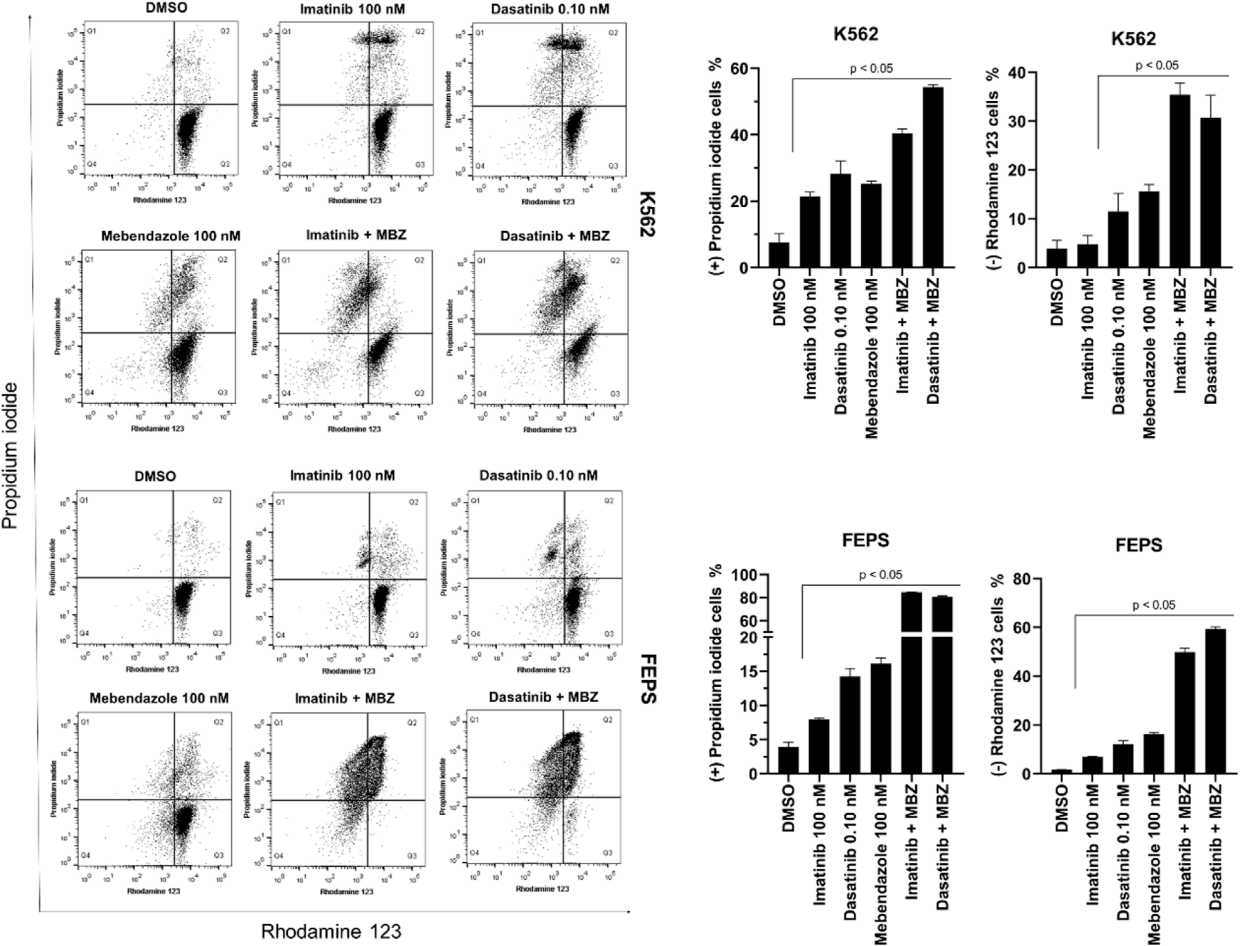
Mebendazole (MBZ) alone and in combination with ABL1 inhibitors induced loss of cell membrane integrity and mitochondrial membrane potential in the chemosensitive and chemoresistant CML cell line. Cells were double treated with dyes propidium iodide and Rhodamine123 after the treatment with imatinib (100 nM), dasatinib (0.10 nM), mebendazole (100 nM), and the combination imatinib (100 nM) with mebendazole (100 nM), and dasatinib (0.10 nM) with mebendazole (100 nM). Data are represented as the mean ± standard deviation of three independent experiments. Significant differences among groups were tested by the two-way ANOVA, followed by the Tukey multiple comparison test.

To understand the mechanism by which MBZ induced death in K562 and FEPS cell lines, the double labeling assay with Rhodamine 123 and PI allowed us to simultaneously analyze plasma membrane permeability and mitochondrial activity of the treated cells (Figure 2). Cells labeled only with Rho123 (PI-/Rho123+) were classified as viable, while those labeled only with PI were cells in late apoptosis or necrosis (PI+/Rho123-). Cells in early apoptosis were classified as an early loss of mitochondrial function (PI-/Rho123-) or an early loss of plasma membrane integrity (PI+/Rho123+) (Figure 2).

K562 cells treated with dasatinib and MBZ alone, as well as all combinations, had approximately 22% and 18% reduction in PI–/Rho123+ labeling (*p* ≤ 0.0001), indicating a reduced viable profile. For MBZ alone and its combination with imatinib, this decrease occurs by an increase of close to 10% of cells in late apoptosis/necrosis (*p* ≤ 0.001). For dasatinib alone and in combination with MBZ, this reduction occurs both by an increase in cells in late apoptosis percentage (close to 14% and 28%, respectively, *p* ≤ 0.0001) and by the increase of depolarized mitochondria cells (close to 15% and 19%) (Figure 2).

For the FEPS strain, as well as for K562, treatments with dasatinib, MBZ alone, and in combinations, including with imatinib, lead to the reduction of viable cells (respectively, ∼6%, *p* ≤ 0.01; 89%, 87%, and 90%, *p* ≤ 0.0001) (Figure 2). For dasatinib treatment, this reduction was 5.5% (*p* ≤ 0.0001), the amount of PI+/Rho123– cells, indicating late apoptosis/necrosis. As for MBZ alone, the number of cells with the apoptotic profile was significantly higher (∼37%) as well as for the two other combinations (38% and 39%, respectively, for combination with imatinib and dasatinib, *p* ≤ 0.0001), but they also have an increase in the number of cells with loss of mitochondrial function (11% and 18%, *p* ≤ 0.0001; Figure 2).

### Pharmacodynamic property prediction

The Molinspiration server was employed to predict the biological activity of molecules studied here by classifying the molecules as inactive (score <−0.5), moderate (−0.5 ≤ score < 0.0), and active (score ≥0.0) for each classification (Table 2). Except for ALB, all molecules including MBZ showed a prediction to be kinase inhibitors (Table 2). Drugs imatinib and dasatinib also showed activity as enzyme inhibitors and GPCR ligand. For other classifications, the molecules were characterized by moderate activity.

**TABLE 2:** Molecule activity score for biological function prediction using the Molinspiration server.

Bioactivity	ALB	MBZ	IMA	DAS	ASC
Kinase inhibitor	−0.04	0.14*	0.59*	0.62*	0.68*
Enzymatic inhibitor	−0.02	−0.05	0.07*	0.05*	−0.09
Protease inhibitor	−0.18	−0.29	−0.08	−0.28	−0.06
GPCR ligand	−0.11	−0.12	0.10*	0.01*	−0.02
Nuclear receptor ligand	−0.62	−0.23	−0.40	−0.59	−0.46
Ionic channel modulator	−0.10	−0.29	−0.09	−0.23	−0.20

*Molecules with the highest activity score. ALB, albendazole; MBZ, mebendazole; IMA, imatinib; DAS, dasatinib; ASC, asciminib.

**FIGURE 3:**
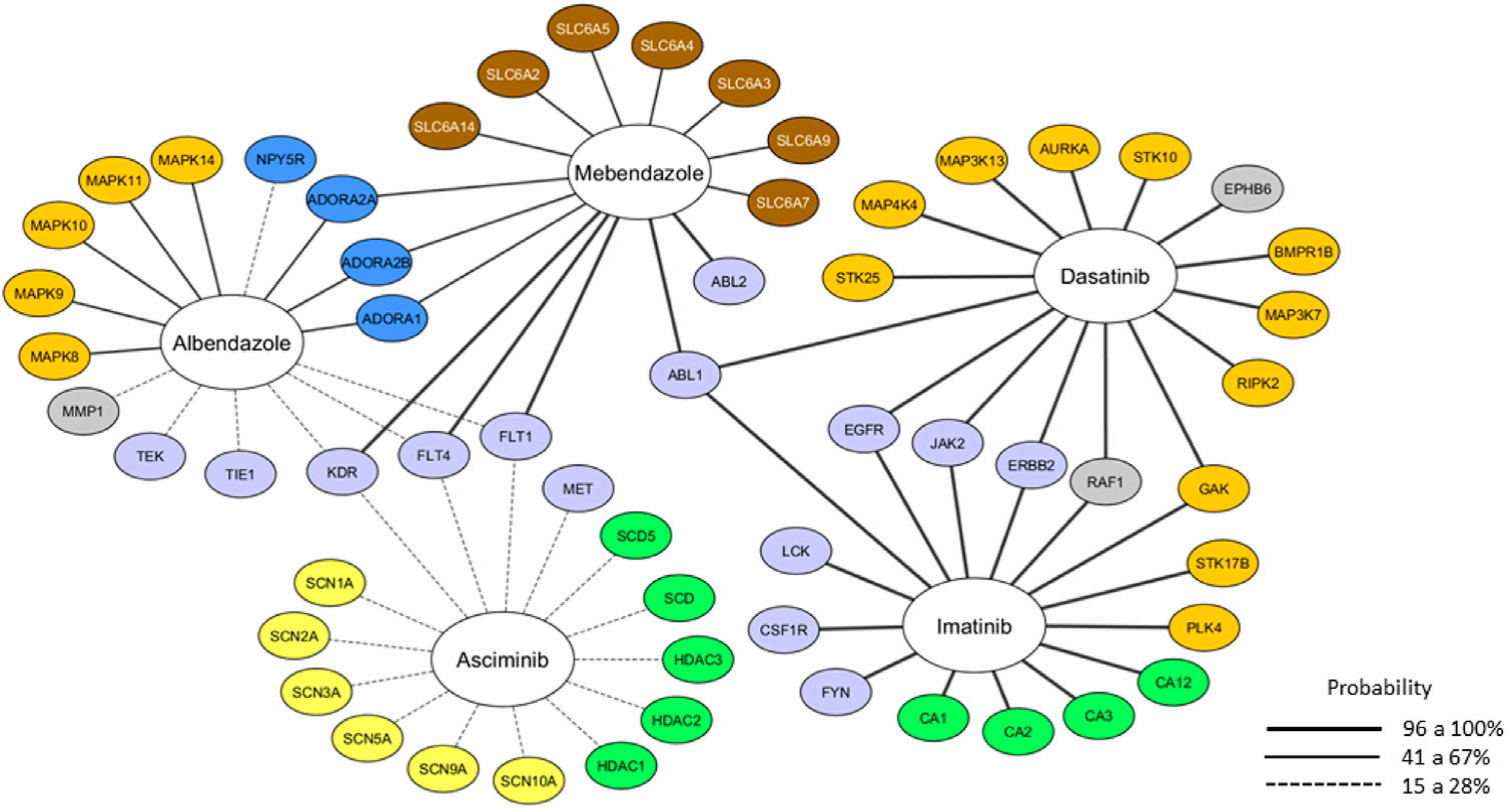
Molecular target prediction using the web server SwissTargetPrediction. The wide line represents the probability of a predicted target being already known one, and the color represents the target class. Light blue: tyrosine-kinase, dark yellow: serine/threonine kinase, green: enzyme, blue: membrane receptor, brown: transporter, yellow: ionic channel, and gray: others.

The SwissTargetPrediction server was employed to perform a target prediction to identify the possible biological targets behind the anticancer activity of MBZ (Figure 3). The analysis compiles all predicted targets, their probability, and classification, and allows the identification of targets in common for studied molecules in addition to MBZ (Figure 3). The results found in the SwissTargetPrediction server agree with those predicted by the Molinspiration server, with 30 of 59 possible targets for MBZ classified as kinases. Among these predicted targets for MBZ, ABL1 showed a high probability of interaction in *Homo sapiens*. Therefore, the MBZ shares the same target as kinase inhibitors used in BCR-ABL1+ CML treatment (imatinib and dasatinib). Together, these results indicated that ABL1 could be a potential target for MBZ based on the ChEMBL database. Then, molecular docking was performed to describe how MBZ could interact with ABL1 compared with canonical inhibitors.

### Mebendazole interacts with ABL1 protein through an allosteric site

**FIGURE 4:**
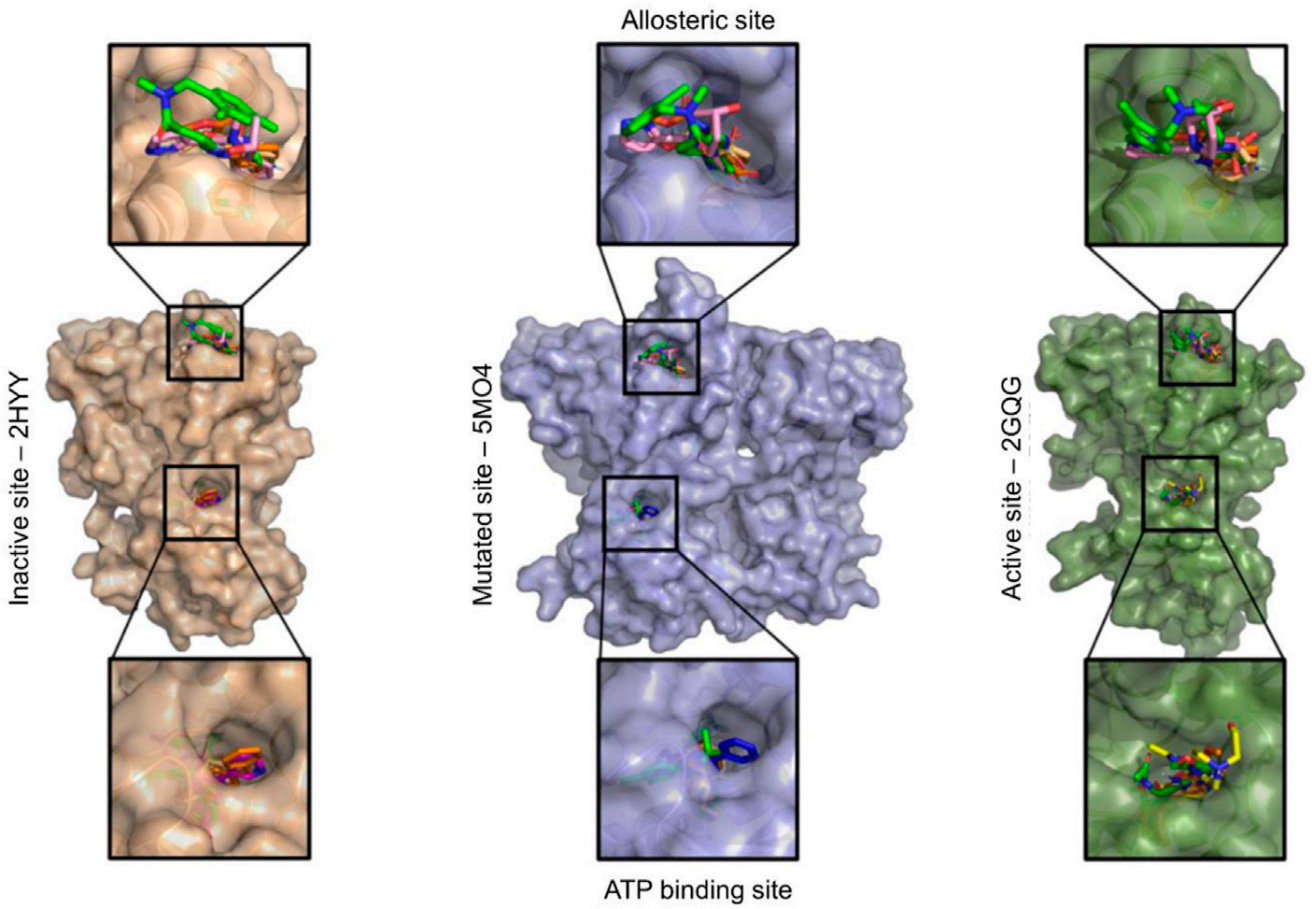
Final ligands’ position (imatinib, dasatinib, mebendazole, albendazole, and asciminib) in the allosteric site and ATP binding site generated by the AutoDock 4.2.1 software.

Given the potential of ABL1 protein to be a molecular target of MBZ, a molecular docking analysis was performed to study the feasibility of interacting between these molecules. Thus, three structures of ABL1 were downloaded from the Protein Data Bank to represent the active (PDB ID: 2GQG) and inactive (PDB ID: 2HYY) conformations, and a third structure presenting the T315I mutation (PDB ID: 5MO4), which is associated with resistance to both imatinib and dasatinib. Once the parameterization of the docking procedure was validated through redocking simulations (RMSD between docked and crystallographic poses ranging from 1.39 to 1.75 Å), MBZ and ALB molecules were docked against both, the ATP binding site and the allosteric binding site. As shown in Table 3 and Figure 4, the resulting interaction energy for MBZ and ALB into the ATP binding site were smaller than those obtained for imatinib, nilotinib, and dasatinib, respectively. Interestingly, when docked into the allosteric binding site, MBZ interacted with more favorable energy than allosteric inhibitor asciminib (Table 4 and Figure 4). It is important to notice that in all structures tested for interaction with the allosteric site, MBZ had a lower calculated IC_50_ (µM) than commercial inhibitor asciminib.

**TABLE 3:** Binding energy (ΔG) and IC_50_ from molecular docking for the ATP binding site of ABL1 protein.

PDB	Ligand	ΔG (kcal mol^−1^)	IC_50_ (µM)	RMSD (Å)
2HYY	Imatinib	−11.95	0.002	1.1015
	Decoy1	−7.06	6.65	-
	Mebendazole	−7.44	3.54	-
	Albendazole	−6.09	34.51	-
5MO4	Nilotinib	−11.25	0.006	0.363
	Decoy2	−8.98	0.26	-
	Mebendazole	−7.10	6.29	-
	Albendazole	−6.88	9.44	-
2GQG	Dasatinib	−8.49	0.603	1.513
	Decoy3	−5.84	52.32	-
	Mebendazole	−6.17	30.16	-
	Albendazole	−5.37	116.61	-

**TABLE 4:** Binding energy (ΔG) and IC_50_ from molecular docking for allosteric site of ABL1 protein.

PDB	Ligand	ΔG (kcal/mol)	IC_50_ (µM)	RMSD (Å)
2HYY	Asciminib	−8.86	0.319	1.750
	Decoy1	−5.50	85.49	-
	Mebendazole	−8.90	0.295	-
	Albendazole	−7.35	4.11	-
5MO4	Asciminib	−6.08	34.88	1.479
	Decoy2	−4.62	411.88	-
	Mebendazole	−6.98	7.69	-
	Albendazole	−6.14	31.84	-
2GQG	Asciminib	−5.97	42.08	1.395
	Decoy3	−3.84	1,530.0	-
	Mebendazole	−7.04	6.95	-
	Albendazole	−6.08	34.93	-

To better characterize the interaction of MBZ with the allosteric binding site of ABL1, we performed a visual inspection in the docking results to identify atomic contacts with closer residues (Table 5). Compared with the allosteric inhibitor, asciminib, MBZ binds to the same residues in the ABL1 protein and reinforces the allosteric action of MBZ (Figure 4). As observed in the crystallographic structure, asciminib forms hydrogen bonds (conventional or carbon) with the residues Gln333, Ala337, Leu341, Tyr435, and Cys464. Interestingly, both asciminib and MBZ presented a consensus for H bonds with residues Ala433 and Glu462. The MBZ also interacts with the Gly463 residue by H bonds.

**TABLE 5:** Residue’s consensus involved in hydrogen bond and hydrophobic interactions for both mebendazole and asciminib in the allosteric site of ABL1 protein.

PDB	Asciminib		Mebendazole	
	Interactions	Distance (Å)	Interactions	Distance (Å)
2HYY	ALA433:O—ASC:H18	2.14	ALA433:O—MBZ:H2N2	1.95
	ARG332:HD1—ASC:O23	2.66	GLU462:O—MBZ:H1C6	2.28
	LEU341:HA—ASC:F	2.52	-	-
	CYS464:H4—ASC:O15	2.62	-	-
	TYR435:OH—ASCH1	3.07	-	-
5MO4	ALA452:O—ASC:H16	2.12	ALA452:O—MBZ:H	2.99
	GLU481:O—ASC:H18	1.8	GLU481:O—MBZ:H	2.07
	LEU360:HA—ASC:F29	2.51	ALA356:HA—MBZ:O	2.82
	LEU360:HA—ASCF30	2.33	GLU481:O—MBZ:H12	2.5
2GQG	GLU462:O—ASC:H18	1.99	ALA433:O—MBZ:H	1.85
	ALA337:HA—ASC:O15	2.48	GLY463:HA2—MBZ:O	2.28
	LEU341:HA—ASC:F	2.09	GLU462:O—MBZ:H13	2.66
	GLU462:OE1—ASC:H7	2.78	GLU462:OE1—MBZ:H13	3.01
	GLU462:OE1—ASC:H8	2.6	-	-

## Discussion

Chronic myeloid leukemia (CML) is a neoplasm with a significant number of new cases of leukemia in adults. CML is estimated to increase the number of cases by more than 60% by 2040, according to a global report (Sung et al., 2021). In addition, CML treatment is a complex challenge given chemoresistance to first- or second-generation drugs (e.g., imatinib and dasatinib) (Jabbour and Kantarjian, 2018). As such, either the development of new therapies or drug repositioning is imperative to assist patients with CML. The development of new drugs is a time- and money-consuming process. Nevertheless, drug repositioning is a great alternative that has gained more attention in the last 2 years, and there was an urgent need to find more effective drugs in a very short time (Sahoo et al., 2021).

In this scenario, computational tools become a good ally to evaluate drugs redirection (Lin et al., 2020). Here, information obtained *in silico* and *in vitro* suggested that the redirection of a benzimidazole, specifically mebendazole, could be a good alternative for CML treatment. Together, *in silico* and *in vitro* approaches enhance drug redirection to discover new therapies. Recently, previous works have already been successful in the redirection of benzimidazoles, to other types of cancer, including another type of cancer, as in the case of MBZ toward human breast cancer (Jubie et al., 2021). In addition, it was shown that the benzimidazole derivatives are not toxic to non-malignant cell lineage models (Nygren et al., 2013; Pinto et al., 2019).

Benzimidazoles have inhibited 50% of cell growth in colon cancer HCT116, RKO, HT-29, HT-8, and SW626 cells at a concentration of 0.5 µM (Nygren et al., 2013), and ∼0.5 µM in gastric cancer AGP01 cells (Pinto et al., 2015, 2019). As far as we know, this is the first study to show the potential of MBZ, a benzimidazole derivate, against chronic myeloid leukemia. Here, MBZ has reached an IC_50_ at a concentration of 104.3 nM and 1917.0 nM, respectively, to K562 and its resistant strain, FEPS (Table 1). Another member of benzimidazoles, albendazole, was not effective against both strains. In addition, the combination of MBZ with tyrosine kinase inhibitors was also an innovation of this work. The combination of the MBZ with the tested inhibitors seems to promote a reduction index of up to ×16, for the combination with dasatinib in the sensitive strain, and up to ×6 if combined with imatinib, for the resistant strain (Table 1). The combination index determined by the Chou-Talalay method demonstrated the synergic effect of MBZ and *BCR-ABL* inhibitors against CML cell lines.

The flow cytometric analysis revealed that MBZ treatment led to an increase in the number of fragmented cells and a reduction of cells in the G0/G1 phase (Figure 2). These results suggest that MBZ (alone and in combination) in nanomolar concentrations promotes cell fragmentation for the K562 characterized by the sub-G1 phase arrest (Figure 2). Results similar to the mechanism of benzimidazole derivatives were described for lung cancer and liver cancer cells (Youssef et al., 2012; Sridhar Goud et al., 2020). Regarding the FEPS strain, it seems that MBZ sensibilizes the FEPS strain to imatinib, causing a large fragmentation accumulation (Figure 2), which did not happen when imatinib was alone. Cytometry analysis also revealed that MBZ induces pro-apoptotic effects, cytoplasmic membrane destabilization, and mitochondrial membrane depolarization in CML cells, which are characteristic events of intrinsic apoptosis (Darzynkiewicz et al., 1994; D’Arcy, 2019).

The *in silico* analysis provided insights into the pharmacokinetic properties of MBZ, such as good intestinal absorption and permeability to the blood–brain barrier, and low toxicity. It is worth highlighting that MBZ is not identified as a p-glycoprotein substrate, which is a characteristic that may contribute to high cytotoxicity (Králová et al., 2013). Likewise, analysis using the Molinspiration server revealed that MBZ presented kinase inhibitory activity (Table 2). In addition, the SwissTargetPrediction server revealed that MBZ interacts with ABL1 and ABL2 proteins (Figure 3). The ABL1 protein is important for CML establishment and is considered a marker for this disease. Based on that, ABL1 has been focused as the target for the development of new inhibitors (Xu et al., 2014; Schoepfer et al., 2018).

Nygren et al. (2013) in their kinome screening showed MBZ activity with Kds in the nanomolar range against ABL kinases. To investigate deeper, the interaction of MBZ with ABL1 molecular docking was analyzed. As it is already known, ABL1 protein adopts active and inactive conformations and has a mutated (T315I mutation) version that is resistant to some drugs. Molecular docking of MBZ and ABL1 was performed in these three situations. In all cases, docking analysis revealed that MBZ binds in an allosteric site far from the active site of ABL1 (Figure 4). After binding in the allosteric site, even far away from the active site, MBZ induces conformational changes impairing the interaction of ABL1 and its substrate, thus inhibiting its activity. This is a good result for three reasons: first, even after the interaction with other molecules (i.e., substrate), MBZ can still interact with ABL1 and inhibit it. Second, by binding in the allosteric site, MBZ makes even hard the development of resistance. For example, the result of T315I mutation and many other mutations emerged during treatment with competitive molecules at the protein’s ATP site, impairing the effectiveness of these drugs. Thus, drugs that target the ABL1 protein allosteric site would be able to maintain the activity against these mutations, in addition to having a superior kinase specificity (Schoepfer et al., 2018). Third, by binding in the allosteric site, MBZ could act synergistically with drugs that target the active site. This result corroborates the synergistic action among MBZ, imatinib, and dasatinib against both K562 and FEPS cell lines of CML (Table 1).

The calculation of binding interaction energy (ΔG) of MBZ to ABL1 revealed that MBZ has a lower energy binding to ABL1 than other FDA-approved drugs targeting ABL1 (Tables 3 and 4). This result indicates MBZ has a higher affinity to interact with ABL1 than commercialized drugs. Interestingly, MBZ also has a high affinity to ABL1 holding the mutation T315I, which is known for conferring resistance to other drugs (Xu et al., 2014). In addition, MBZ presented a lower concentration of IC_50_ to interact with ABL1 than those FDA-approved drugs. Altogether, these results suggest the idea that MBZ has a higher affinity to interact with ABL1 than commercial drugs. This result highlights the potential of MBZ as a new drug targeting ABL1 and thus being used in CML clinics.

In recent years, numerous clinical studies with the use of mebendazole in patients with different tumor types, such as gastrointestinal tumors and central nervous system tumors, are being developed, and in none of them, the combined or isolated MBZ administration showed any side effects to the patients of these studies (Gallia et al., 2020; Mansoori et al., 2021). Mansoori et al. (2021) evaluated the mebendazole prescription of 4 g/day for 30 days in patients with refractory gastrointestinal tumors. None of the patients presented adverse reactions or side effects; however, no clinical improvement was observed, suggesting the need for further studies to assess a better response. In this sense, the data demonstrated in our work point to the rational use of the combined therapy with mebendazole and TKIs for a dosage that can be followed along with the standard clinical prescription for CML treatment, which will not compromise the patient’s safety and may enhance the efficiency of their therapeutic response.

## Conclusion

The results presented here suggest drug repositioning of MBZ to treat chronic myeloid leukemia could be a good option for patients given the efficiency, *in vitro*, of MBZ against both sensible and resistant strains of CML. Although MBZ interacts with a common target in CML, the ABL1 protein, its interaction is different from that of the common drugs. MBZ interacts allosterically in a position far away from the active site. This ability of MBZ might support the synergistic effect of it with other drugs, helping to overcome CML resistance to drugs generating perspective for future work with other tyrosine kinase inhibitors. This pioneering study revealed the anticancer potential of MBZ against CML either alone or in combination with other drugs. In addition, it is important to highlight the applicability of MBZ in the clinic, given that the drug is already commercialized and well accepted by patients.

## Data Availability

The original contributions presented in the study are included in the article/Supplementary Material; further inquiries can be directed to the corresponding author.
